# Successful treatment for sports hernia by total extraperitoneal repair with intraperitoneal examination: Report a case

**DOI:** 10.1016/j.amsu.2022.104954

**Published:** 2022-11-17

**Authors:** Toshikatsu Nitta, Jun Kataoka, Masatsugu Ishii, Yasuhiko Ueda, Masato Ohta, Ryo Iida, Takashi Ishibashi

**Affiliations:** Division of Surgery Gastroenterological Center, Medico Shunju Shiroyama Hospital, Osaka, Japan

**Keywords:** Sportsman's hernia, Sports hernia, Total extraperitoneal repair

## Abstract

**Introduction:**

and importance: TEP might be one of options for treating such a sports hernia.

**Case presentation:**

An 18-year-old Japanese male presented with right groin pain for approximately two years. The pain was initially felt on the right side only, especially on kicking. We assessed the patient using laparoscopic examination with an intra-abdominal scope and subsequently diagnosed a sports hernia with a bilateral internal inguinal hernia. We then performed total extraperitoneal repair (TEP) for its treatment. The patient had a good postoperative course and was discharged from our hospital in remission after 3 days. Finally, the patient was able to play soccer without groin pain.

**Clinical discussion:**

Chronic groin pain in athletes can be caused by a bulge in the posterior inguinal wall, consistent with an incipient direct inguinal hernia.

**Conclusion:**

We show that intraperitoneal examination with TEP might be one of options for treating such a sports hernia. Endoscopic placement of the retropubic mesh must be considered an important option for this type of hernia.

## Highlights

• We assessed the patient using laparoscopic examination with an intra-abdominal scope and subsequently diagnosed a sports hernia with a bilateral internal inguinal hernia.
• We show that intraperitoneal examination with TEP might be one of options for treating such a sports hernia.
• Endoscopic placement of the retropubic mesh must be considered an important option for sports hernia.

## 1 Introduction

Sports hernia is regarded as a cause of chronic groin pain for athletes. The management of chronic pain in athletes was complicated and the diagnosis of sports hernia was said to be difficult.

In sports hernia (sportsman's hernia/athlete's hernia/athlete's pubalgia), surgical repair of the posterior inguinal wall, according to the IEHS guidelines, often exhibits excellent results [1]. Additionally, the endoscopic placement of a retropubic mesh is said to be more effective than conservative therapy for the treatment of sports hernia.

In this report, the indication for surgery was groin pain. This was hypothesized to be caused by a weak posterior inguinal wall. The syndrome of weakness of the posterior inguinal wall without a clinically recognizable hernia causing chronic groin pain has not been widely appreciated. However, this syndrome can be treated surgically with excellent results [2].

Herein, we introduce a strategy that involves intraperitoneal examination for diagnosis, followed by total extraperitoneal repair (TEP) for treatment of sports hernia. We believe that our laparoscopic management of this type of case could be effective for chronic groin pain.

Written informed consent' was obtained from the patients and legally authorized representatives for anonymized patient information to be published in this case report. This case has been reported in line with the SCARE 2020 criteria [3].

## 2 Case presentation

An 18-year-old Japanese male complained of right groin pain for approximately two years. He had been playing football at a junior and high school club for the last six years. He initially felt pain on the right side of the groin, particularly while kicking a ball. He could not participate in a football game without pain but had no complaints regarding activities of daily life.

He had consulted many hospitals and clinics. And he was performed physical examination and ultrasound and computed tomography (CT) and magnetic resonance imaging (MRI), but the cause of this pain was nuknown. As a result he had been suffering from this pain and taking a pain killer which is not effective.

**Fig. 1:**
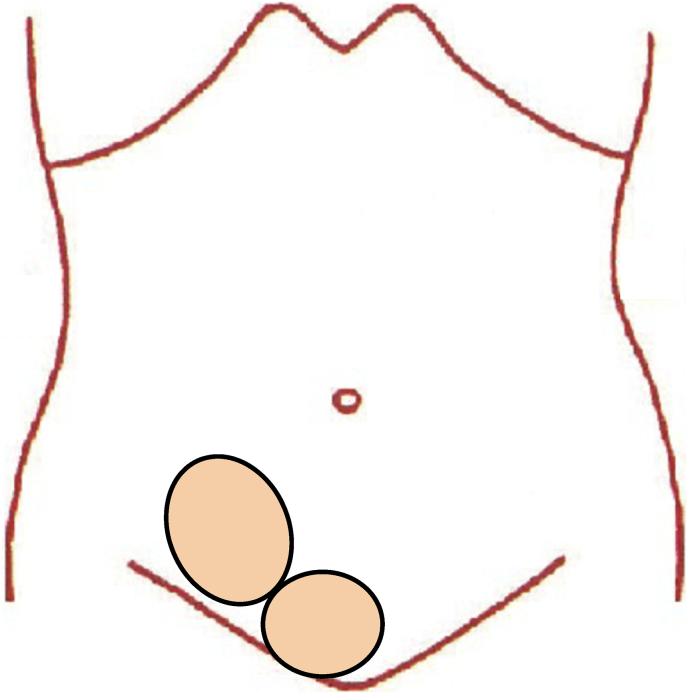
Diagram to enable patient to identify areas where pain was felt. The pain was worse on the right side but extended across the midline and involved the rectus abdominis muscle and pubic bone.

**Fig. 2:**
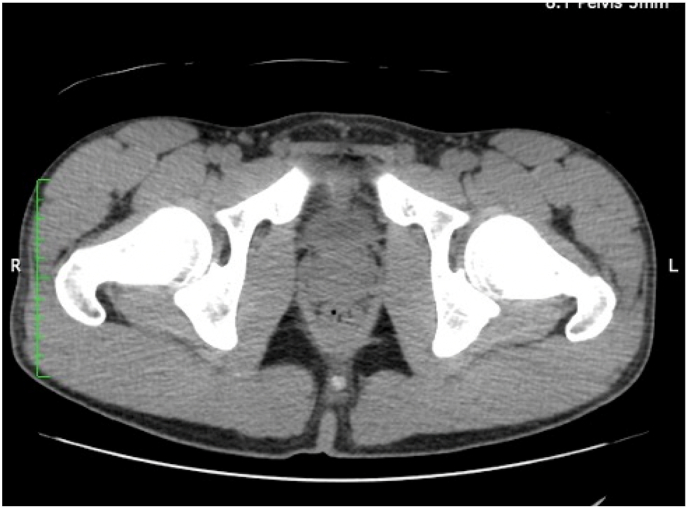
Abdominal enhanced computed tomography. Enhanced computed tomography (CT) of the abdomen showed no sign of inguinal hernia.

Although the pain was worse on the right side, it extended across the midline and involved the rectus abdominis muscle and pubic bone (Fig. 1). There was no obvious swelling or injury mark on the bilateral inguinal wall, and no inguinal hernia was seen upon physical examination, no lipoma was seen on Ultrasound. Additionally, enhanced computed tomography (CT) of the abdominal region showed no signs of inguinal hernia (Fig. 2). Based on these findings, the patient was admitted to the Department of Surgery at Shiroyama Hospital. He was suspected a sports hernia and was scheduled for laparoscopic examination with the possibility of laparoscopic hernioplasty if the hernia was identified.

**Fig. 3:**
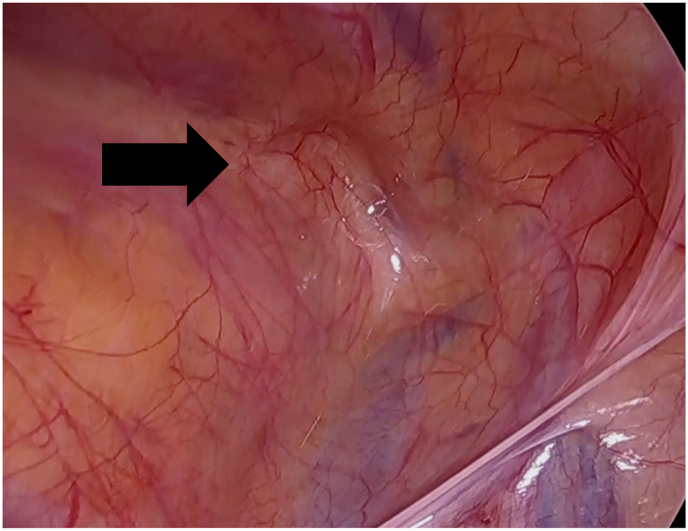
Laparoscopic examination of the right side. Intraoperative findings on the right side pertain to direct hernia（black arrow）.

**Fig. 4:**
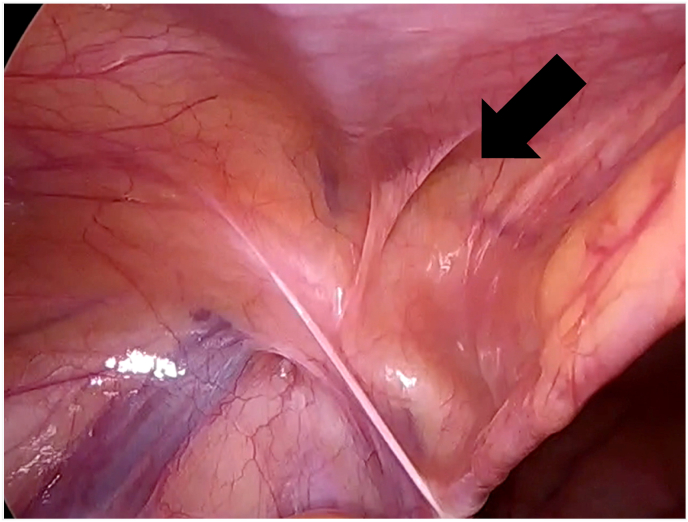
Laparoscopic examination of the left side. Intraoperative findings on the left side pertain to direct hernia（black arrow）.

Surgery was performed with the patient under general anesthesia and in the supine position. A port was placed 12 mm below the umbilicus at the midline, and two working ports were placed in the midline between the umbilicus and the pubis. The lower port was placed two finger breadths above the pubis symphysis (i.e., the three-port method). We determined the presence of a bilateral inguinal hernia with laparoscopic examination using an intra-abdominal scope [4,5]. In this case, findings on the right (Fig. 3) and left (Fig. 4) side pertained to direct hernia. The size of right direct hernia measured as 2 fingers (M2)and its left as 1 finger (M1) according to the EHS groin hernia classification [6]. Thus, a sports hernia with bilateral internal inguinal hernia was diagnosed.

We dissected the Retzius space inside the epigastric arteriovenous dissections were made through a subumbilical incision without a balloon. Lateral dissection of the preperitoneal space was performed. We isolated cord structures (parietalization), and PROGRIP™ (COVIDIEN) was subsequently placed in the preperitoneal space and tacking was accomplished using AbsorbaTack™ (COVIDIEN). The total operative time was 150 min, and the intraoperative blood loss was <5 ml. The patient had a good postoperative course and was discharged from our hospital in remission after 3 days. After over 6 months of follow-up, no pain was observed and the patient was able to play soccer without groin pain. Written informed consent was obtained from the patient for publication of this case report and accompanying images.

## 3 Discussion

Groin pain in athletes, particularly in Australian rules football and soccer players, is a common occurrence. Chronic groin pain, as a result of sports hernia, is a difficult diagnosis as it often develops gradually and with uncharacteristic symptoms. We recognize a chronic groin syndrome in which the patient has variable pain distribution, which is aggravated by exercise, coughing, sneezing, and sit-ups [7].

Sports hernia is known to cause maximal tenderness, painful cough impulse, and operative findings suggesting distension of the posterior inguinal canal wall musculature — an early type of direct hernia [2]. Chronic groin pain in athletes may be due to a bulge in the posterior inguinal wall, consistent with an incipient direct inguinal hernia. Therefore, chronic groin pain in sportsmen caused by a distension of the posterior inguinal wall is effectively described as an early direct inguinal hernia.

Hackney et al. [2] have attributed chronic groin pain to stretching and tearing of the transversalis and conjoint tendons. The first implicates a reduction in internal rotation of the hip joint. Inward twisting then produces a shearing force across the pubic symphysis from the pull of the adductor [8,9]. This leads to stress on the musculature of the inguinal wall, perpendicular to the fascia and muscle fibers. Stretching of the transversalis and tearing from the inguinal ligament account for the pain.

The posterior position of the mesh behind the conjoint tendon and pubic bone creates a stronger repair than anterior mesh placement. Laparoscopic repair, such as transabdominal preperitoneal (TAPP) and total extraperitoneal (TEP), appear to be as effective as conventional anterior repair for sports hernia [10]. Nevertheless, diagnosis is important, as the key to successful surgery is whether or not a groin hernia is present. We suggest performing an intraperitoneal examination for the diagnosis and treatment. Therefore, TAPP is suitable for sports hernia compared with TEP with regard to intraperitoneal examination, but TEP is an especially effective option for the treatment of bilateral hernias [11].

The superiority of the TEP repair over nonoperative therapy has already been shown in an Randomized Controlled Trial [12]. And there is no data regarding TAPP. We believe that intraperitoneal examination with TEP is effective for treating sports hernias. Additionally, endoscopic placement of the retropubic mesh must be considered a serious option for sports hernias, according to the IEHS guidelines [1].

The etiology of sports hernia is almost completely explained; however, without obvious groin herniation, it is important to consider how the sports hernia should be treated. Future work should consider conservative treatment of refractory sportsman hernia and radiofrequency denervation of both the ilio-inguinal and inguinal ligaments in the short term, as an alternative to anesthetic and steroid injection as stated by the IEHS guidelines [1]. Regardless, the whenever chronic groin pain in athletes occurs and the cause could be unknown by other examination such as physical examination and ultrasound, CT, MRI, intraperitoneal examination with TEP should be performed to check for bilateral early direct hernia.

## 4 Conclusion

TEP might be one of options for bilateral inguinal hernia, but intraperitoneal examination is also necessary, especially unknown chronic groin pain in athletes.

## Ethical approval

We have gotten he ethical approval of this study by ethics committee.

Unique identifying number or registration ID: Reserch Registry 8124.

Hyperlink to your specific registration (must be publicly accessible and will be checked):

Shiroyama 2018-004.

（https://www.shiroyama-hsp.or.jp/patient/cancer/ethics.html）

## Please state any sources of funding for your research

None of the authors has any conflict of interest to declare.

## Author contribution

We believe that this surgical report is unique and educational.

All authors engaged in the therapy of this patient.

Obviously we surgeons peformed this operation as a team.

This teman comination of our hospital could perform theses therapies.

## Registration of research studies

1. Name of the registry: Surgical strategy for sports hernia
2. Unique identifying number or registration ID: Reserch Registry 8124
3. Hyperlink to your specific registration

## Guarantor

Author Toshikatsu Nitta

Guarantor is Takashi Ishibashi who is president of Shiroyama Hospital and my supervisor.

## Consent

We were explained to the patient and relatives, and informed consent was obtained.

And we submit the certification as a guarantor.

Written informed consent was obtained from the patient for publication of this case report and accompanying images. A copy of the written consent is available for review by the Editor-in-Chief of this journal on request.

## Provenance and peer review

Not commissioned, externally peer-reviewed.

## Annals of medicine and surgery

The following information is required for submission. Please note that failure to respond to these questions/statements will mean your submission will be returned. If you have nothing to declare in any of these categories then this should be stated.

## Declaration of competing interest

No conflict of interest to report.
